# The Support Needs of Patients Requesting Medical Aid in Dying and Their Relatives: A Qualitative Study Using Semi-Structured Interviews and Written Narratives

**DOI:** 10.3389/ijph.2024.1606878

**Published:** 2024-11-28

**Authors:** Stijn Vissers, Joni Gilissen, Joachim Cohen, Luc Deliens, Freddy Mortier, Kenneth Chambaere, Sigrid Dierickx

**Affiliations:** ^1^ End-of-Life Care Research Group, Department of Family Medicine and Chronic Care, Vrije Universiteit Brussel, Brussels, Belgium; ^2^ End-of-Life Care Research Group, Department of Public Health and Primary Care, Universiteit Gent, Ghent, Belgium; ^3^ Department of Philosophy and Moral Sciences, Bioethics Institute Ghent, Universiteit Gent, Ghent, Belgium

**Keywords:** euthanasia, assisted dying, support needs, patients, relatives

## Abstract

**Objectives:**

To explore the support needs that patients and relatives experience throughout their medical aid in dying (MAID) trajectories.

**Methods:**

A qualitative study in Belgium in 2022 using 1) semi-structured interviews with and personal written narratives of patients requesting MAID and 2) semi-structured interviews with relatives of patients requesting MAID. We performed a qualitative content analysis.

**Results:**

We included in our analysis the lived experiences of 15 patients and 21 of their relatives. We identified eight types of support needs: support for 1) maximizing daily functioning (only reported by patients), 2) making sense of the unbearable suffering (only reported by relatives), 3) managing meaningful activities, 4) navigating existential questions, 5) psycho-emotional regulation, 6) facilitating social interaction, 7) understanding the process toward MAID, 8) and handling organizational and practical matters.

**Conclusion:**

Patients and relatives might experience multidimensional support needs throughout their MAID trajectories. Our findings suggest that they experience these trajectories more as social/existential pathways than as medical ones. A palliative care approach may be an effective way to fulfill the support needs of patients and relatives throughout their MAID trajectories.

## Introduction

Medical aid in dying (MAID), including euthanasia and physician-assisted suicide, has become an increasingly important legal and medical issue. Euthanasia involves the practice in which a health practitioner ends a person’s life at their voluntary request by administering lethal drugs. Physician-assisted suicide involves the practice in which a health practitioner provides or prescribes to a patient, at his or her voluntary request, a lethal medication which the patient subsequently self-administers to end their own life [1]. At present, jurisdictions with MAID legislation cover about 300 million people across the globe [1]. Furthermore, prevalence rates of MAID continue to rise in jurisdictions where the practice is lawful [2–4]. Several jurisdictions are currently debating a possible enactment of MAID legislation.

MAID entails a complex process as patients requesting it and their relatives may experience a range of challenges, difficulties and undesired outcomes throughout their MAID trajectories [5]. These trajectories include the moment when the patient expresses an explicit desire for MAID, the assessment of the request, the performance of MAID, and the period following the performance of MAID [5, 6]. Patients, for example, sometimes feel that their autonomy and values are not respected, and may feel that healthcare professionals and relatives minimize their unbearable suffering [5, 7, 8]. Relatives, for example, may feel unprepared for the performance of MAID, can experience post-traumatic stress after the performance, and may feel excluded by healthcare professionals during the assessment of the MAID request [9–14]. These challenges, difficulties, and undesired outcomes may indicate that the support needs of patients and relatives are not adequately fulfilled, for instance, because their healthcare professionals do not know their support needs or have not assessed them properly [6, 7, 9, 15].

The existing scientific literature provides little direction on the specific support needs of patients and relatives throughout their MAID trajectories. Studies have primarily focused on describing their general experiences with MAID practice, their attitudes towards MAID, problems and difficulties they encounter in MAID practice, and their interactions with healthcare professionals [8, 16]. A better understanding of the support needs of patients and relatives in MAID practice could guide healthcare professionals in anticipating undesired outcomes and improving the quality of life of patients and relatives [17]. We sought to answer the following research question: what support needs do patients requesting MAID and their relatives experience throughout their MAID trajectories?

## Methods

### Research Paradigm and Study Design

We took on a social constructionist lens, assuming that support needs are social and dynamic constructs [18]. Due to the explorative nature of our research aim, we employed a qualitative design capturing the lived experiences of MAID practice among patients and relatives, using 1) semi-structured interviews with patients and relatives and 2) personal written narratives from patients through qualitative questionnaires.

In this study, we defined the medical aid in dying trajectory as beginning when the patient expresses an explicit desire for medical aid in dying, followed by the assessment of the request, the performance of medical aid in dying, and the period following the performance of medical aid in dying [5, 6].

### Study Context

We conducted this study from December 2021 to September 2022 in Flanders and Brussels (Belgium). For a patient to be eligible for MAID, specific criteria must be met which are stipulated in the Belgian Act on Euthanasia [19]. In Belgium, medical aid in dying is mainly performed in the home setting (54% of all registered cases in 2020–2021), in hospitals (30%), and in nursing homes (13%) [20].

### Research Participants, Recruitment, and Data Collection

We included patients and relatives having lived experiences with MAID practice. Eligibility criteria for patients were: 1) having expressed a desire for MAID to relatives (friends or family members) or healthcare professionals, or having formally requested MAID to an attending physician, or having received the formal approval from an attending physician to receive MAID; 2) being comfortable with being interviewed in Dutch; 3) residence in Flanders or Brussels, Belgium; and 4) being 18 years or older. Eligibility criteria for relatives of patients were: 1) being a family member or friend of a person who has expressed a desire for MAID or has formally requested MAID, or who has received the formal approval to receive MAID, or who received MAID prior to the interview (between 3 months and 3 years), 2) fluency in Dutch; 3) residence in Flanders or Brussels, Belgium; and 4) being 18 years or older.

We recruited participants between December 2021 and June 2022 using purposive and snowball sampling. We aimed to recruit a heterogeneous study sample in terms of sociodemographic characteristics, health conditions and principal care settings. More specifically, purposive sampling occurred through healthcare and patient organizations and associations, stakeholders, and the professional network of the End-of-Life Care Research Group in Flanders (Belgium). Snowball sampling was applied by inviting potential participants who were identified by those already included for participation in the study. Interested participants could register on a website or contact the research team. We conducted an eligibility assessment before inviting them to participate in the study. Eligible patients were allowed to participate via a semi-structured interview or a personal written narrative using an online qualitative questionnaire (LimeSurvey). We ensured that the interviewer was unfamiliar to the participants. Prior to study participation, we disclosed the identity of the interviewer to participants. Moreover, we offered participants the opportunity to get in touch with the interviewer for any questions or concerns regarding the study.

For the semi-structured interviews, we used a topic guide consisting of the following topics: 1) general experiences with MAID practice; 2) experienced practices regarding MAID; 3) experienced good practices regarding MAID, and 4) experienced support needs regarding MAID. The specific questions in the topic guide were iteratively refined throughout the data collection process. Support needs were conceptualized as the support or guidance a participant experienced as essential to meet his or her basic needs or to achieve a desired outcome in MAID practice [21]. Participants could choose whether the interview would be performed online by Whereby (http://Whereby.com) or in person at a location of their choice. XX (second author, M.Sc. in Social work, Ph.D. in Health Sciences, senior researcher, female) conducted one interview, while XX (first author, M.Sc. in Sociology, Ph.D. candidate, male) conducted the other interviews. Both are researchers with previous experience in conducting qualitative health research. Interviews were recorded and transcribed verbatim. After every interview, field notes were made to document unique observations and contextual information. For the personal written narratives, we sent an online qualitative questionnaire (LimeSurvey) to eligible patients who preferred this method of study participation [22]. The questionnaire included open-ended questions reflecting the themes of the semi-structured topic guide. We requested patients to respond to these questions by writing down their narratives and experiences, which we converted into transcripts for data analysis [22]. Data collection was informed by inductive thematic saturation in which we defined the saturation point as the stage at which no new meaning related to support needs emerges across the narratives of patients and relatives [23].

### Data Analysis

We used a qualitative content analytic approach [24, 25]. In the first phase, XX and XX (last author, M.Sc. in Sociology, Ph.D. in Health Sciences, senior researcher, female) applied open inductive coding and categorization of the raw data, analyzing patients’ and relatives’ experiences separately. We linked initial codes to text fragments, aiming at identifying initial codes describing participants’ lived experiences of support needs regarding medical aid in dying. Support needs were conceptualized following the definition used during data collection [21]. XX and XX independently created a coding framework based on seven transcripts. These frameworks were then compared; inconsistencies and differences were discussed until a preliminary coding framework was reached. Next, XX used the coding framework to analyze the remaining transcripts, modifying it when new codes emerged. We used Nvivo 12 for the coding process. In the second phase, XX merged codes similar in understanding into meaningful clusters to identify types of support needs. Moreover, we concluded that saturation was reached after the 11th interview for patients and the 18th for relatives, as no new meaning in relation to support needs had arisen [23]. In the third phase, the identified clusters were discussed in group meetings with all co-authors. In this third phase, we also concluded that most types of support needs of patients are identical to those of relatives, with only a few different types. For clarity in reporting, the latter are described separately in the results section of this article. Again, inconsistencies were addressed until an agreement on final meaningful clusters, i.e., types of support needs, was reached. The field notes were used to help us organize the codes and identify meaningful clusters.

In terms of reflexivity, the research team included four health sociologists (SV, SD, KC, JC, and LD), one social worker (JG), and one bioethicist (FM), all with prior experience in qualitative methods related to end-of-life practices. None of the researchers hold strong normative positions for or against medical aid in dying. Each team member adopted a critical perspective, advocating for rigorous evaluation of medical aid in dying legislation and practices. To uphold objectivity in data interpretation, the research team convened regular discussion sessions.

### Ethical Considerations

The Ethics Committee of the XX and XX approved our study (XX; 15 September 2021). We utilized pseudonyms for all participants in the transcripts and removed any identifying information. All participants provided written or oral informed consent to participate in the study.

## Results

### Study Sample

We identified 18 patients and 23 relatives as eligible for study participation, all of whom were invited to participate. 15 patients and 21 relatives (n = 36) eventually participated, whereas the other invited individuals chose not to participate or to drop out without providing their motives. We conducted nine semi-structured interviews with patients (five online interviews and four in person at the patient’s main residence), one dyadic semi-structured interview with a patient and a relative (in person at the main residence of the patient), and 20 semi-structured interviews with relatives (11 online interviews and 9 interviews in person at the main residence of the relative), and included five written narratives from patients. The mean length of the semi-structured interviews with patients was 78 min (range: 28–130 min), and with relatives 82 min (range: 54–127 min). The main characteristics of the participants are listed in Tables 1, 2.

**TABLE 1:** Participant characteristics of patients requesting medical aid in dying (N = 15) (Belgium, 2021–2022).

	N
Biological sex	
Female	12
Male	3
Age	
<30 years	1
30–40 years	4
41–50 years	4
51–60 years	3
>60 years	3
Highest level of education	
Secondary school	6
Higher education	9
Phase in the MAID trajectory^a^	
Holding an explicit desire for MAID that has been expressed to relatives or healthcare professionals	4
Exploration of the eligibility for MAID by the attending physician following a formal request	5
Preparing the performance of MAID as the attending physician has agreed to grant the request	6
Main medical diagnosis	
Psychiatric disorder	7
Connective tissue disorder	2
Chronic pain syndrome	2
Neurodegenerative disorder	2
Cancer	2
Main residence^a^	
Home	13
Hospital	1
Nursing home	1

MAID, medical aid in dying.

^a^ Timing of the interview or written narrative.

**TABLE 2:** Participant characteristics of relatives (N = 21) (Belgium, 2021–2022).

	N
Sex	
Female	18
Male	3
Age	
<30 years	1
30–40 years	6
41–50 ears	2
51–60 years	6
<61 years	6
Highest level of education	
Secondary school	2
Higher education	19
Relationship with the patient	
Partner	2
Parent	3
Sibling	1
Child	9
Grandchild	2
Close friend	1
Living together with the patient^a^	
No	18
Yes	3
Family carer^a^	
Yes	11
No	9
The phase of MAID practice^a^	
The patient holds an explicit desire for MAID, that has been expressed to relatives or healthcare professionals	0
Exploration of the eligibility for MAID by the attending physician following a formal request	2
Preparing the performance of MAID as the attending physician has agreed to grant the request	3
MAID was carried out^b^	
<12 months	7
12–24 months	5
25–36 months	3
>36 months	1
Patient’s main medical diagnosis^a^	
Cancer	12
Neurodegenerative disorder	3
Psychiatric disorder	2
Chronic pain syndrome	2
Polypathology	2
Patient’s sex	
Female	13
Male	8
Patient’s age^a^	
<30 years	1
30–40 years	2
41–50 years	1
51–60 years	2
61–70 years	4
71–80 years	7
>80 years	4
Patient’s main residence^a^	
Home	14
Hospital	4
Nursing home	3

MAID, medical aid in dying.

^a^ Timing of the interview or when MAID was carried out.

^b^ In one case, the relative did not attend the performance of MAID.

### Support Needs Experienced Throughout the Medical Aid in Dying Trajectory

**FIGURE 1:**
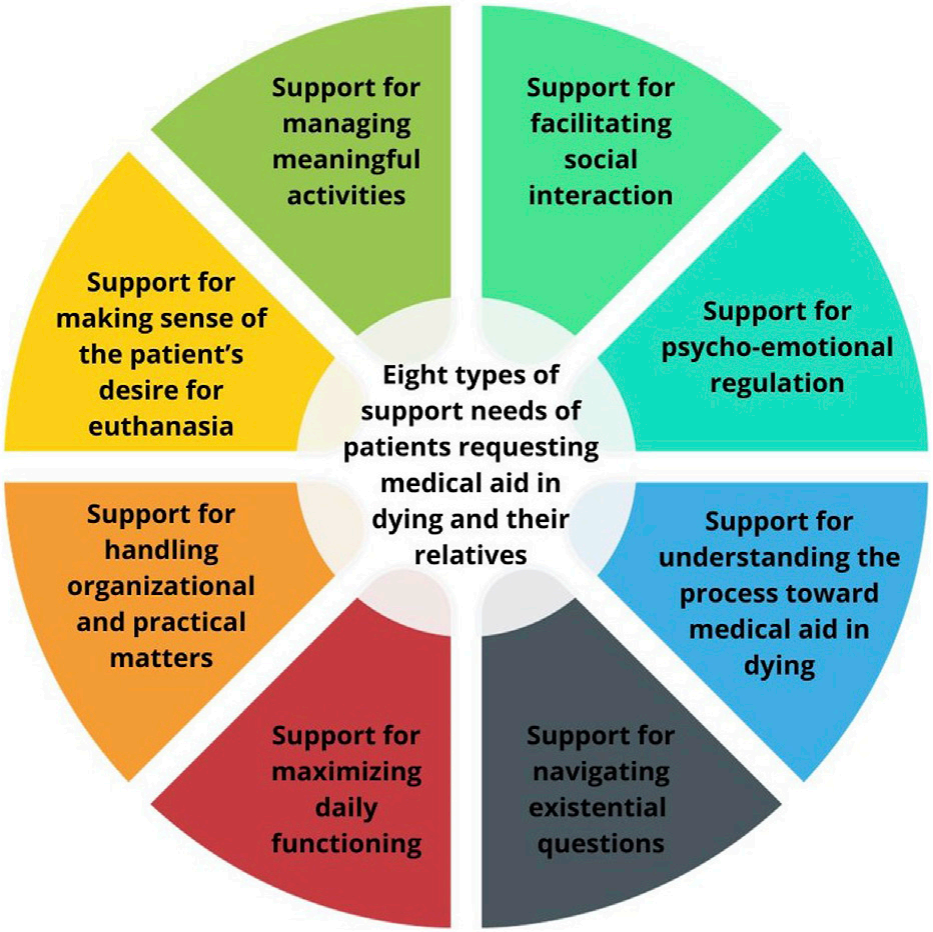
Eight types of support needs were identified that patients and their relatives might experience throughout their medical aid in dying trajectories. Support for maximizing daily functioning was only reported by patients, while support for making sense of the patient’s desire for medical aid in dying only in relatives. (Belgium, 2021–2022).

We identified eight types of support needs experienced by patients and relatives throughout their MAID trajectories (Figure 1). Identified support needs appeared to differ in their intensity of experience and seemed to be partially linked to a specific phase of the MAID trajectory, e.g., assessment or day of performance. We discuss each theme or type of support need separately for analytic clarity. However, they are interconnected in practice. In the following paragraphs, references to “participants” include both patients and relatives. Furthermore, some exemplary quotes are provided in Table 3.

**TABLE 3:** Participant’s quotes regarding types of support needs (Belgium, 2021–2022).

Support for maximizing daily functioning
*“Well, you won’t recover if you have a chronic disease. So it’s not about recovering. It’s a different way of looking at me. I also know that, in my case, being at home is not the ideal situation because I don’t have the same support as in the hospital. There, you got pain management, a nursing team helping you out of bed and giving you all the medication, and if they have to, even putting the food in your mouth. I don’t need that kind of support. That is way too much, of course. But just a bit more support, so I am more comfortable at home. This would be helpful, so I can stand on my own feet and stay here at home with my dog until it’s time for my MAID.”* - Patient preparing the performance of MAID-
Support for making sense of the patient’s desire for MAID
*“I think it would have been better if we were a bit more informed about her suffering, and especially about the severity of her suffering. Of course, that’s very personal, but that would have helped us to evaluate her situation in a realistic way and perhaps to assist her better. A bit like raising awareness. Now, I can finally imagine what her suffering was all about, but before … Mum makes the decision for MAID and stops all treatments. Two weeks later, she was dead, and we were still unsure of why she had chosen to go through with MAID.”* -Relative of a patient who received MAID-
Support for managing meaningful activities
*“I would prefer to call it the need for a master of ceremonies. Perhaps that’s a weird word, but someone who actually guides us in our farewell ritual. When I say farewell ritual, everyone thinks of the farewell ritual in the mortuary. So I don’t need him for that, but for a farewell ritual on the day of the performance, together with my family. Someone who helps you in looking for alternative ways to say goodbye. Someone who normalizes the whole situation on the day itself, and in which my brother and mother are given a special part.”* -Patient preparing the performance of MAID-
Support for navigating existential questions
*“I also think the philosophy around it is important. What does this mean in a more existential context? I am not religious, but what does such a MAID request from your child mean? What is the impact of that? Have I been a good parent? Is it something genetic? What was my role in her decision to make the request? It’s not just the practical side that matters, but also, how do I deal with this? What does it mean? Should I ask myself questions about how I stand in life? These questions are ever-present. Fortunately, I don't get completely overwhelmed by these questions, otherwise, I would spiral into depression. However, I still contemplate them regularly. I have numerous questions, observations, and reflections that are difficult to be answered. It’s a state of mind that persists even when I am in bed at night. This can lead to mental turmoil, making it impossible to get any sleep. So yeah, then it would be nice to have someone to listen to them and acknowledge my concerns. That could provide some comfort, yes.”* -Relative of a patient whose attending physician is assessing the eligibility for MAID-
Support for psycho-emotional regulation
*“That moment itself [after performance], you are in the flow of the funeral and family. But the night after, it was, it was really awful. Yes, you are in shock. When someone you love dies due to a sudden accident or illness, you are in shock because you have not been able to prepare for it. We had a year to prepare ourselves for her death as we had arranged her MAID well in advance. But I was in shock. […] A month later, I ended up with a psychologist through my general practitioner. I didn't know what to do with what I felt or didn't feel. Is it normal for me to think such things? And then someone who says to you ‘That’s completely normal’ is just enough.”* -Relative of a patient who received MAID-
Support for facilitating social interaction
*“It would be useful to have someone as a backup, indeed. I have always had a good relationship with my brothers, you know. One of them is Catholic, and the others are atheistic. Anyway, when we are together, that can cause some problems, and even more now when I have asked for MAID. Sometimes, you can really feel the tension. But you can’t always be diplomatic if that is what I want. But it would help if there is a moderator. We often meet as brothers, but we don’t really talk to each other about our feelings. That’s difficult. So yes, a moderator to help us say what we still want to say to each other before I get MAID.”* –Patient whose attending physician is assessing the eligibility for MAID-
Support for understanding the process toward MAID
*“Perhaps the oncologist could have framed it better: from the moment you decide that you want MAID, this and that still have to happen first. We didn't know that. Mom called the hospital to say she would like to receive MAID. All the necessary paperwork was in order. However, at that point, she was told that a second doctor was needed. We also didn't know that you couldn't come into the hospital and have it done right away.”* -Relative of a patient who received MAID-
Support for handling organizational and practical matters
*“One thing that was lacking in terms of support during my hospital stay was the absence of social service to help me with the arrangements necessary for the performance of MAID. I had to carry out everything on my own, without any prior experience of making a written request, knowing whom to notify, or what to take into account. That was a time-consuming and energy-draining process, and it would have been helpful if there was a social service to rely on. Someone who could be present when arranging these things; and also help me locate a notary in the area. The practical details that needed to be taken care of were a burden, and while those around me have tried to help me, I don’t think it should have been their responsibility to shoulder this.”* -Patient preparing the performance of MAID-

MAID, medical aid in dying.

#### Support for Maximizing Daily Functioning

This support need was only found in patients. Patients repeatedly spoke of their unbearable suffering as a daily reality around which they had organized their lives. They described in many ways their ongoing struggle with accepting this suffering. Although patients recognized that no practical support could eradicate their suffering, some felt that support could ease their struggle by maximizing their daily functioning throughout their MAID trajectories. They favored practical support in carrying out daily activities and tasks, e.g., aid to travel to healthcare facilities. Some patients experienced such support as essential to maintaining a sense of self-worth. On the other hand, others experienced such support as pivotal to being able to stay at home independently and to receive MAID in their familiar environment.

#### Support for Making Sense of the Patient’s Desire for MAID

This support need was only identified in relatives. Relatives needed support for making sense of the patient’s desire for MAID and its broader context, such as the nature of the unbearable suffering. Most relatives needed this to 1) facilitate closure and accept the patient’s desire and 2) evaluate their potential role in alleviating the suffering. Relatives stated that this support need partially stemmed from their limited understanding of the desire for MAID as patients and healthcare professionals often reduced the desire to the patient’s medical condition. Moreover, relatives indicated that this support need was particularly strong in the early phases of the MAID trajectory, when they first learned of the patient’s desire or had just become involved in the process.

#### Support for Managing Meaningful Activities

Patients and relatives (hereafter referred to as participants) felt the need to experience moments and activities that gave purpose and meaning to their MAID trajectories as ways to make the most of the remaining time prior to the performance. In practice, these meaningful moments and activities reflected participants’ values, passions, lives, roles, and identities, particularly those of patients. These included, for example, preparing memorials, sharing (life) stories and memories, and participating in rituals and traditions. Participants particularly experienced a farewell ritual as one of the most meaningful activities. Moreover, relatives also identified being a family carer for the patient in his or her MAID trajectory as a meaningful activity. However, participants needed support to manage meaningful activities as they often faced difficulties and challenges. Additionally, patients with a non-terminal condition most often found their MAID trajectories to be long and purposeless and stipulated that they required support in finding meaningful activities prior to the performance.

#### Support for Navigating Existential Questions

Participants expressed having many existential questions and contemplating life and death throughout their MAID trajectories. This involved questioning and reflecting upon, for example, the value of life, when it is justified to request MAID, what will come after death, whether requesting MAID equals failing relatives (experienced by patients), or when one is ready to die. Consequently, participants emphasized their support need for navigating existential questions as like having a sounding board since these questions sometimes led to feelings of loneliness and distress. Furthermore, this support need appeared more acute in patients than in relatives.

#### Support for Psycho-Emotional Regulation

A rapid succession of negative and positive emotions characterized participants’ MAID trajectories. They particularly stressed the need for guidance in controlling and coping with negative emotions. Participants often felt overwhelmed by the intensity of their emotions and faced difficulties processing them individually. Patients’ negative emotions included anxiety due to the uncertain outcome of the MAID request, distress caused by the unpredictable progression of the unbearable suffering, and fear of death and the unknown afterlife. Negative emotions of relatives included feelings of loss and anticipatory grief throughout their MAID trajectories, stress when the request was being assessed, and tension upon receiving the news of the patient’s request.

The need for psycho-emotional regulation seemed to differ in intensity among participants. In their accounts, participants experiencing adverse events in their MAID trajectory emphasized their need for psycho-emotional regulation more strongly compared to those not experiencing adverse events. Examples of adverse events included witnessing the patient’s discomfort, complications or delays in accessing MAID, and communication issues with providers. Additionally, family carers or relatives very close to the patient also stressed their need for psycho-emotional regulation more firmly in their accounts compared with others. These relatives attributed this need in part to the fact that they neglected their emotions due to prioritizing the patient’s emotions as the focal point throughout the MAID trajectories.

#### Support for Facilitating Social Interaction

Participants felt a strong need for social connection and sharing their MAID trajectories with those close to them, who were often indicated as the most important individuals in their trajectories. However, many participants reported problems establishing and maintaining social interactions, and wished for support to facilitate this.

The support need for facilitating social interaction was materialized by participants in several ways. First, some participants required support to resolve social conflicts and to improve social bonds, e.g., conflict resolution through moderated family conversations. This support need was partly related to obtaining peace of mind before the performance. Moreover, some patients considered postponing the performance if social conflicts were not resolved. Second, a few patients needed social support in communicating their MAID desires or requests to relatives. Third, some relatives perceived this support as facilitating family engagement in the MAID trajectories. These relatives experienced family engagement as necessary to adequately support patients themselves in their MAID trajectories. Relatives who were at times not being engaged, experienced frustration or anger, sometimes resulting in psychological problems.

#### Support for Understanding the Process Toward MAID

In their MAID trajectories, participants required support for understanding the process toward MAID regarding 1) the MAID legislation and procedure, and 2) the attending physician’s decision-making regarding MAID. First, participants found it challenging to comprehend the legal modalities of MAID, especially the eligibility criteria. These difficulties led to confusion and ambiguity, such as whether patients with advanced dementia have access to MAID via an advance directive. In addition, participants wanted guidance in understanding the MAID procedure, including the formal steps one must complete before the performance. Second, participants required guidance in understanding the attending physician’s decision-making process for granting or rejecting the MAID request. Participants wanted to know the specific motives or reasoning behind the decision, partly due to their experiences of vagueness from attending physicians.

#### Support for Handling Organizational and Practical Matters

Participants required assistance with various organizational and practical matters throughout their MAID trajectories, such as deciding on the ideal location and timing of performance or arranging the patient’s funeral. They also needed help with administrative tasks, e.g., completing a written MAID request to meet the legal requirement.

Participants explained that this support need partly stemmed from their desire to focus on issues that mattered more to them than organization and practical matters, such as social activities. Organizational and practical support was perceived as a way to reduce the administrative burden often experienced in MAID practice. On the other hand, some patients wished for this support to be in order with all administrative requirements as a sense of control or relief. That was more acute in relatives than patients because handling organizational and practical matters was perceived as one of their important roles since patients often lacked strength due to their general deterioration.

## Discussion

Using semi-structured interviews and written narratives, we identified various support needs among patients and relatives across their MAID trajectories i.e., from the moment when the patient conceives an explicit desire for MAID till the period following the performance of MAID. Participants needed support for maximizing daily functioning (only reported by patients), making sense of the patient’s desire for MAID (only reported by relatives), managing meaningful activities, navigating existential questions, psycho-emotional regulation, facilitating social interaction, understanding the process toward MAID, and handling organizational and practical matters.

Our study suggests that MAID constitutes a multidimensional practice for patients and relatives involving various support needs throughout their MAID trajectories. These support needs essentially reflect a much broader aspiration of patients and families for definition and experience of the conditions for a good death [26–29]. Although echoing some degree of specificity in terms of context and practice – e.g., making sense of the patient’s desire for MAID and understanding the process toward MAID-most identified support needs seem to correspond to the multidimensional support needs of patients and relatives in other end-of-life trajectories [30–32]. Our findings could be seen to align with Manfred Max-Neef’s theory which states that (support) needs across individuals and practices are highly similar, as opposed to traditional theories asserting considerable variation in (support) needs [33]. Hence, an inter-professional approach can be warranted to meet the multidimensional support needs throughout the MAID trajectory as it can be challenging for a single professional to address all of these needs alone.

Our study illustrates that, in line with other dying experiences, patients and relatives experience their MAID trajectory less as a medical practice and more as a social and personal process [34, 35]. This is substantiated by our finding that participants needed support for facilitating social interaction while emphasizing their intense need for social connection and sharing their final moments throughout their MAID trajectories. The social character of MAID practice is also evident in relatives’ need for support to be engaged in the MAID trajectories, making them active participants. These results build on previous studies indicating that MAID should be equally understood and approached as a social and relational phenomenon [8, 12, 36]. This implies that the MAID trajectories of patients and relatives are partly nurtured by the dynamics and quality of their relationships, which may influence their MAID experiences considerably [12, 36]. In that way, our findings provide empirical evidence for the suggestions of Canadian scholars that MAID support would benefit from both a patient- and family-centered approach [7, 13, 37]. Furthermore, “MAID as an existential practice” is substantiated by the fact that participants needed support for navigating existential questions, managing meaningful activities, and making sense of unbearable suffering in the case of relatives. This is in keeping with the study of Tuva et al., which found that patients near the end of life longed for care focusing on “living a meaningful life” [30]. Furthermore, these support needs may result from the typical existential experiences that are often reported among patients and relatives when death is imminent [38–40]. Moreover, our participants experienced farewell rituals in their medical aid in dying trajectories as particularly meaningful. That corroborates previous research, in which relatives perceived these farewell rituals as an advantage of medical aid in dying practice as they experienced that in other end-of-life practices, they would have less room to organize them due to the unpredictable nature of the moment of death [12, 41]. Successful farewell rituals may provide individuals with emotional energy and facilitate closure [41, 42]. Furthermore, our study suggests that patients and their relatives struggle to organize and manage meaningful and meaning-making activities. A plausible reason is that such tasks might come with an emotional burden and some unfamiliarity [5].

Our study further raises the interesting question about which support model would be best suited to meet the support needs of patients and relatives in medical aid in dying practice. In line with others [7, 43, 44], we argue that a palliative care approach seems to be highly suitable, based on our findings’ alignment with the principles and goals of palliative care: 1) patients requesting medical aid in dying and relatives require support for the multidimensional needs they experience with a strong emphasis on psychological, social and existential needs; 2) an inter-professional approach is advisable to fulfill the multitude of these support needs adequately; and 3) patient-and-family centeredness appears the most appropriate approach. These requirements mean that palliative care professionals and services are well suited to address the support needs of patients and relatives in medical aid in dying practice. Thus, palliative care and medical aid in dying practice should not necessarily be viewed as opposing options but might be integrated, as in Belgium [45]. The integration of palliative care and medical aid in dying practice continues to be the subject of intense international debate [46, 47]. Our study provides empirical support in favor of this integration. We suggest that jurisdictions with (proposed) assisted dying legislation should consider in earnest to what extent and how palliative care professionals can be engaged, and organizations can be strengthened, to support patients and relatives throughout their medical aid in dying trajectories.

### Limitations and Strengths

With regard to study limitations, first, some psychological adjustments and recall bias regarding earlier experiences in the medical aid in dying trajectories may be possible (e.g., because of memory limitations and emotional influences), resulting in reporting predominantly positive or negative experiences. Recall bias, may have been possible, for example, for those relatives who were included in the study during their bereaved phase or after the administration of medical aid in dying. Second, we could have missed some specific support needs of patients with terminal conditions as we mainly recruited patients with non-terminal conditions. We mitigated this by including numerous medical aid in dying experiences from relatives of patients with terminal conditions. On the other hand, various study strengths can be acknowledged. We used several strategies to enhance the rigor and trustworthiness of our findings: method triangulation, investigator triangulation, and ongoing reflection [48]. We also used a combination of semi-structured interviews and written narratives. The latter method of data collection was presented at initial contact with patients to include those more difficult to engage with an interview format, aiding inclusivity and reducing potential recruitment bias. We obtained a saturation of types of support needs arising from the lived experiences of both patients and relatives.

### Implications for Clinical Practice and Future Research

First, our findings offer valuable insights that can inform healthcare professionals, educational curricula, practice tools –e.g. guidelines for medical aid in dying- and training-programs about which support needs can improve the medical aid in dying experiences and outcomes of patients and relatives. Secondly, our study provides empirical evidence for key components that should be included in a needs-oriented approach to care for or support medical aid in dying practice, such as a person-centered approach. Thirdly, healthcare professionals should pay attention to the non-medical support needs of patients and relatives, with particular attention to their social and existential needs. In this regard, promising approaches to facilitating social interaction between patients and relatives throughout their medical aid in dying trajectories may include family therapeutic methods, family group discussions, and nurse-delivered dyadic interventions [49–53]. Following this, future research could investigate the most effective type of support to fulfill the social and existential support needs of patients and relatives in medical aid in dying practice, given the current lack of research on this topic.

### Conclusion

Our study reveals that patients and relatives might experience multidimensional support needs throughout their medical aid in dying trajectories. Our findings suggest that they experience their medical aid in dying trajectories as explicitly social/existential in nature. As their support needs essentially correspond with those experienced in other trajectories at the end of life, support for patients and relatives in medical aid in dying practice might benefit from a palliative care approach.
